# Localised Effects of a Mega-Disturbance: Spatiotemporal Responses of Intertidal Sandy Shore Communities to the 2010 Chilean Earthquake

**DOI:** 10.1371/journal.pone.0157910

**Published:** 2016-07-06

**Authors:** Roger D. Sepúlveda, Nelson Valdivia

**Affiliations:** 1 Instituto de Ciencias Ambientales y Evolutivas, Facultad de Ciencias, Universidad Austral de Chile, Valdivia, Chile; 2 Centro de Investigación: South American Research Group on Coastal Ecosystems (SARCE), Universidad Simón Bolívar, Caracas, Venezuela; 3 Instituto de Ciencias Marinas y Limnológicas, Facultad de Ciencias, Universidad Austral de Chile, Valdivia, Chile; 4 Centro de Investigación FONDAP: Dinámica de Ecosistemas Marinos de Altas Latitudes (IDEAL), Universidad Austral de Chile, Valdivia, Chile; National Taiwan Ocean University, TAIWAN

## Abstract

Determining the effects of unpredictable disturbances on dynamic ecological systems is challenged by the paucity of appropriate temporal and spatial coverage of data. On 27 February 2010, an 8.8 Mw mega-earthquake and tsunami struck central Chile and caused coastal land-level changes, massive damage to coastal infrastructure, and widespread mortality of coastal organisms. Wave-exposed sandy beaches showed significant changes of species abundances from before to after the earthquake, but the highly dynamic biotic and abiotic conditions of these habitats make difficult to draw clear-cut conclusions from these patterns. Here, we analysed a beyond-BACI (Before-After Control-Impact) sampling design to test whether the effects of the Maule earthquake on sandy-shore species diversity, abundance, and structure were heterogeneous along the shore. Invertebrate species abundances were quantified before (i.e. February 2010) and after (i.e. March 2010, September 2010, and March 2011) the earthquake at three sandy shores randomly located within the earthquake rupture area and three sites within a “control” area located >400 km southward from epicentre. Immediately after the earthquake took place, the three sites located in the rupture area showed anomalous beach-profile uplifts that did not comply with the erosion (i.e. “negative” uplifts) that regularly occurs during late summer in the region. Species richness, abundance, and community structure significantly varied from before to after the strike, but these patterns of change varied among sites within both areas. Only the site with the strongest and persistent beach-profile uplift within the rupture area showed significant concomitant changes in species richness and community structure; after 13 months, this community showed a similar multivariate structure to the before-disturbance state. This site, in particular, was located in the section of the rupture area that received most of the impact of the after-earthquake tsunami. Therefore, our results suggest that the effects of the Maule mega-earthquake on the ecological communities were spatially heterogeneous and highly localised. We suggest that high mobility and other species’ adaptations to the dynamic environmental conditions of sandy beaches might explain the comparatively high resilience of these assemblages. With this work we hope to motivate further experimental research on the role of individual- and population-level properties in the response of sandy-beach communities to interacting sources of disturbances.

## Introduction

Understanding the patterns of species diversity, abundance, and community structure after natural and anthropogenic disturbances is central to provide accurate predictive models of community recovery and resilience. However, lack of before-impact and spatially extensive data usually hampers our ability to infer causality of patterns. In addition, the effects of disturbances on local communities can be difficult to determine in systems characterised by high spatiotemporal variability in environmental conditions and population abundances [1]. Patterns in natural abundances depend on the spatial scale of observation [2], so that significant responses to disturbances may be discernible at certain scales, but not necessarily at others [3,4]. Therefore, appropriate temporal and spatial coverage at multiple scales is necessary to assess the magnitude of disturbances affecting natural communities.

In coastal ecosystems, mega-disturbances such as hurricanes and earthquakes can generate massive events of mortality in intertidal and shallow subtidal communities. The Mw 8.8 mega-earthquake and tsunami that struck central Chile on 27 February 2010 generated coastal land-level changes, sand deposition, and mortality of coastal organisms at a number of locations along the shores [5,6]. The epicentre of this earthquake, the fifth largest ever instrumentally recorded in the world, was located ca. 120 km northeast of Concepción (36°49’S, 73°03’W); the maximum length of rupture was bounded between 34°S and 38°30’S, resulting in a rupture area of ca. 500 km long [7,8]. Within this area, co-seismic land-level changes were heterogeneous along the shore, and the largest uplifts were recorded in Arauco Peninsula (37.1°S to 37.7°S). In Isla Santa María (37°02’S, 73°31’W), for example, coastal uplift caused massive mortality of intertidal rocky-shore sessile species [5]. Moreover, the tsunami waves following the earthquake deposited large quantities of sediment on sandy beaches along the shore, changing the slope, width, and sedimentary characteristics of beaches to varying degrees [6]. Accordingly, post-stroke changes in intertidal sandy-shore species abundance were heterogeneous along the rupture area, and depended on particular features of sites such as land-level change, height wave, and local coastal infrastructure [6].

Along the rupture zone, most of the coast is characterised by wave-exposed sandy shores [9]. These habitats usually show sharp seasonal changes in physical conditions, because winter storms can remove large amounts of sand from the beach, hold them in suspension, and then deposit them offshore [10,11]. This swell-related erosion result leads to a decrease in beach profile of wave-exposed sandy shores during late summer and throughout winter (see ref. [6] for southern Chile). Sandy beaches therefore have been regarded as dynamic and varying environments, where organisms must be very mobile and able to burrow before being swept offshore by the swash [11]. In fact, it is expected that sandy-beach species are extraordinarily adapted to habitat changes and physical instability. Sandy-shore populations, moreover, can be highly dynamic over the year. For example, dominant intertidal invertebrates can show contrasting patterns of temporal variation, from apparently stochastic to clearly seasonal [12]. Therefore, assessing the potential impact of unpredictable disturbances—such as the Maule earthquake—on sandy-shore communities is challenged by a high spatiotemporal variation in both, physical settings and local population abundances.

Beyond-BACI (Before-After Control-Impact) designs are powerful tools usually used to assess the impact of anthropogenic disturbances on local populations in variable environments [13,14]. These designs can be used, therefore, to accurately determine the degree to which the Maule earthquake and tsunami impacted the local assemblages in the dynamic wave-exposed beaches in southern Chile. Sandy shores cover more than the 70% of the ice-free coasts of the world, are used for several recreational activities, and are probably the best-known marine environment to most humans [11,15]. These coastal habitats are highly vulnerable to climate change-related impacts, especially erosion caused by increased storms and sea-level rise [16,17]. Despite the relevance and vulnerability of these shores, the understanding of community-wide responses to disturbances in these systems has lagged behind in development compared to other marine systems [15,18].

Here, using a beyond-BACI sampling design, we explore the short- to mid-term patterns of intertidal sandy shore assemblages before and after the Maule earthquake. We hypothesise that, since the earthquake caused a heterogeneous land-level change within the rupture area [7,19], the concomitant change in local assemblages should follow a heterogeneous spatial pattern. Therefore, we predict that (1) the Maule earthquake generated significant changes on sandy-shore species diversity, abundance, and structure, and that (2) those impacts were stronger at those sites showing larger morphodynamic changes.

## Materials and Methods

### Ethic statement

In this study, no specific permits were required to access the sampling sites, because these are unrestricted to public access, are not privately owned, and not designated as protected areas. No protected or endangered species were involved in this study.

### Study area and sampling design

**Fig 1:**
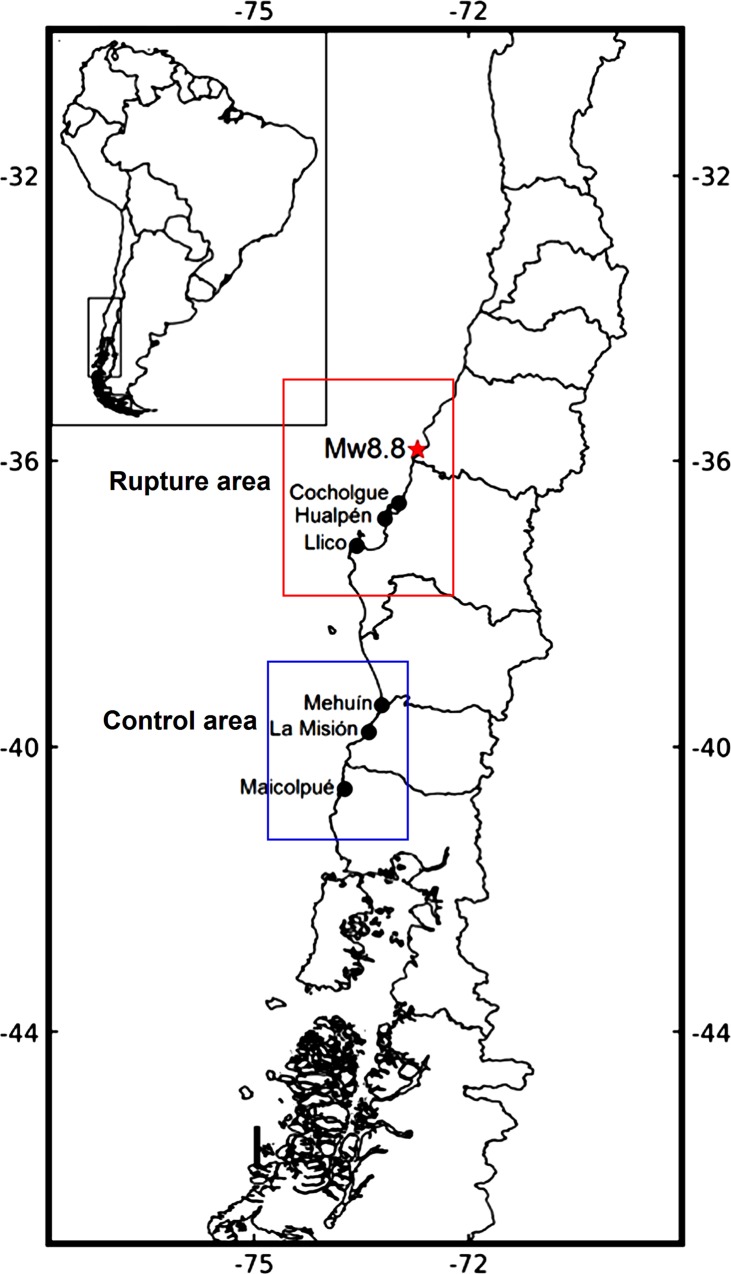
Map of the sampling sites in the central Chilean coast. The Maule earthquake epicentre is indicated by a red star. Cocholgüe, Hualpén, and Llico were located within the earthquake rupture area (red area) and <150 km of lineal distance to epicentre (∼36°S). Mehuín, La Misión, and Maicolpué were located within the “control” area (blue area) and >400 km away from the epicentre (∼40°S).

This study took advantage of an on-going sampling programme of intertidal sandy-shore assemblages arranged along the central Chilean coast (~36°S to 40°S, Fig 1) during early February 2010; that is, before the Maule earthquake took place in 27 February. Samplings of several times before the disturbance would have been desirable in order to have a more comprehensive picture of the community-level responses. Unfortunately, this was not feasible due to the stochastic nature of earthquakes. After the earthquake, species abundances were estimated in March 2010, September 2010, and March 2011.

The multi-scale sampling programme encompassed two areas of ~200 km in alongshore length (Fig 1): a first area around the Maule earthquake epicentre (hereafter referred to as rupture area) and a second area located >400 km south of the epicentre (hereafter referred to as “control” area). Within each area, three intertidal sandy-shore sites were randomly located; Cocholgüe, Hualpén, and Llico sites were located in the rupture area, and Mehuín, La Misión, and Maicolpué sites were located in the control area (Fig 1). At each sampling site and time, five replicate transects spaced from one another by 10 m were extended from high to low tidal heights. Along each transect, ten core samples of sediment were collected with a metallic corer of 0.1 m diameter and buried 0.3 m depth at equally distances across the intertidal zone (= 0.002 m^3^ per corer), which were integrated in one sample to obtained 0.023 m^3^ of sediment per transect. The sand was screened through a 1-mm sieve mesh to collect all macroinfauna, which were preserved in a 70% Ethanol solution until further taxonomic classification. In the laboratory, the organisms where sorted and identified to the lowest taxonomic level (usually species) that could be achieved on the basis of morphological differences observable with a binocular microscope. Species abundances were expressed as individuals per transect (i.e. ind./0.023 m^3^). The number of taxonomic identities (i.e. species richness) and the sum of all species abundances (i.e. total abundance) were calculated from the species abundance dataset (S1 Table in the Supporting Material). In addition, we estimated the difference in species richness and total biomass from before to after the stroke took place. This was done by randomly pairing transects from before (February 2010) and immediately after the earthquake (March 2010), and then estimating the mean pairwise difference for each sampling site.

Finally, we used the Emery’ method to elaborate a profile of the beach at each site. The beach profiles were repeated every sampling time in order to determine the morphological dynamics of the intertidal sandy shores. Then, the before-after earthquake difference in beach height was estimated for each distance from coastline and for each site.

### Data analysis

Permutational Multivariate Analysis of Variance (PERMANOVA, [20]) were used to analyse separately species richness and total abundance based on Euclidean distances matrices from raw data, and community structure based on Bray-Curtis dissimilarities matrix from fourth-root transformed data. PERMANOVA was performed using “Time” (four levels: February 2010, March 2010, September 2010, and March 2011) and “Area” (two levels: rupture and control areas) as fixed factors, and “Site” (six intertidal rocky shores) as random factor nested within “Area”. In order to test our predictions and generate appropriate *a priori* independent contrasts, we identified the site(s) with stronger profile deformations after the earthquake. Llico, within the rupture area, was the only site showing a discernible pattern of deformation over time (see Results: Spatiotemporal changes in beach morphology, below). Therefore, two independent contrasts were included in the statistical models: a “temporal contrast” comparing before (February 2010) vs. after (the March 2010-September 2010-March 2011 centroid) the earthquake, and a “spatial contrast” comparing Llico (Ll) vs. the mean response of Cocholgüe and Hualpén (C-H) within the rupture area. A significant interactive effect of the temporal contrast by Area would support the prediction (1) that the Maule earthquake generated significant changes on sandy-shore species diversity, abundance, and community structure; a significant effect of the spatial contrast by Time would support the prediction (2) that the impact of the Maule earthquake was stronger at those sites showing larger geomorphological deformations. Multivariate spatiotemporal patterns in species abundances were depicted by means of Principal Coordinates Analysis (PCoA) based on Bray-Curtis dissimilarities calculated from fourth-root transformed data [21]. All probability values were derived from a pseudo-F distribution calculated through 10,000 permutations of the same dataset; when the simulated permutations were <1000, the probability value was obtained through Monte-Carlo simulations. Both, uni- and multivariate analyses were performed using PERMANOVA+ for the PRIMER statistical package.

## Results

### Spatiotemporal changes in beach morphology

**Fig 2:**
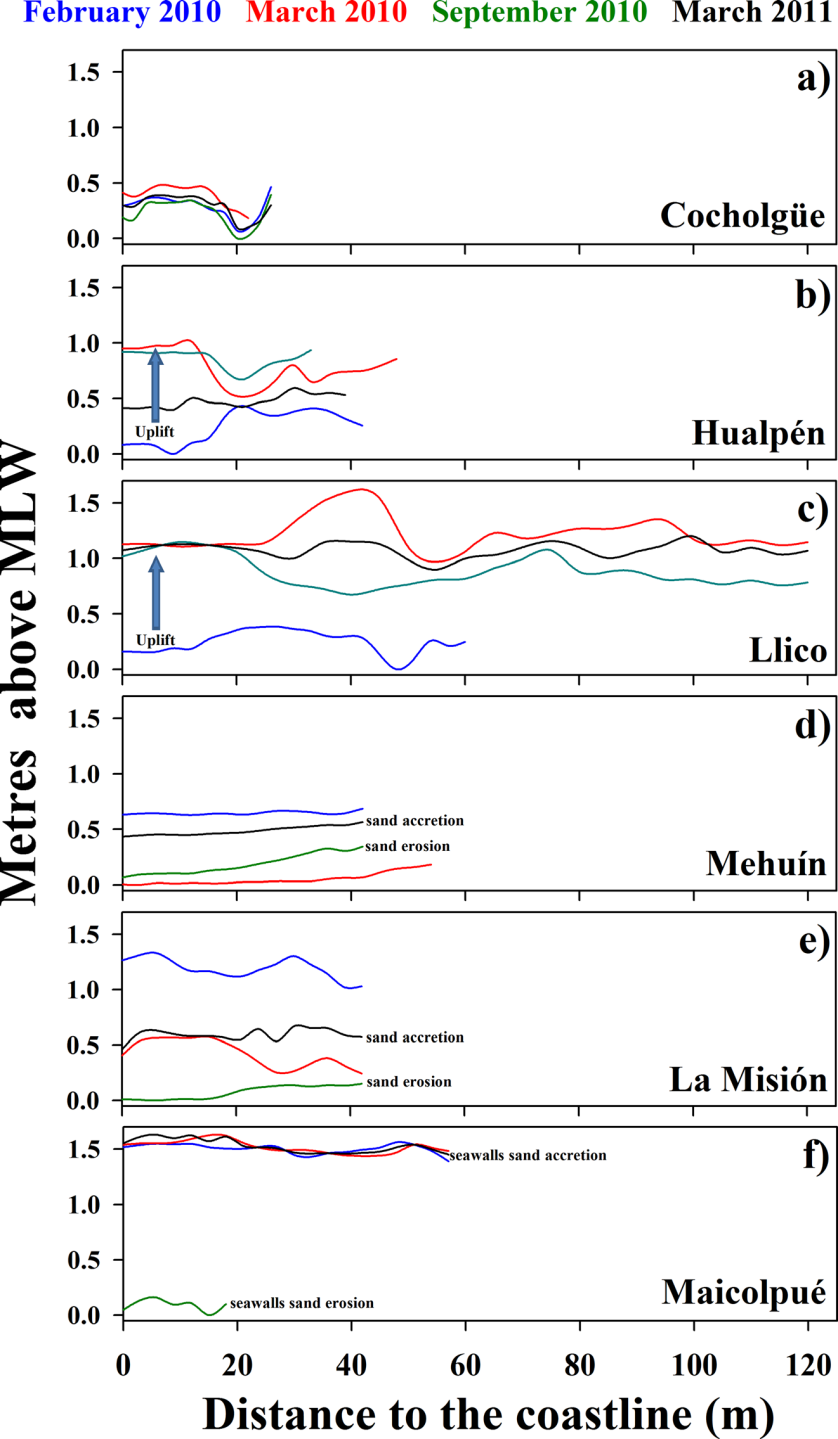
Profile of beaches in the earthquake rupture and control areas. For each site, each line represents the profile of the sampling beach at each sampling time. “Metres above MLW” (mean low water) denote the height of the beach at a given distance from the coastline. Profiles are given for before (February 2010, blue line) and after (March 2010, red; September 2010, green; and March 2011, black line) the earthquake and tsunami of February 27, 2010.

The six study sites showed different spatiotemporal patterns in beach profile. Within the rupture area, the three shores showed different degrees of beach-profile uplifts, contrary to the regular downward movement of beach profiles during late summer. The beach-profile uplift at Cocholgüe (northern site, Fig 2A) was small and shore morphology remained relatively stable over time. Hualpén and Llico, on the other hand, performed height changes up to 0.95 m from February to March 2010 (Fig 2B and 2C; compare the vertical positions of blue and red lines). In addition, beach width at Llico (Fig 2C) increased from ca. 0.20 m in February 2010 to 1.20 m in March 2010. Within the control area, Mehuín and La Misión decreased in height during the sampling period (Fig 2D and 2E), according to the process of erosion that normally starts on late summer. In Maicolpué, we observed a single reduction in beach height and width during September (austral spring) 2010; the beach returned to its previous morphology by late austral summer in March 2011 (Fig 2F).

### Ecological responses of community to the Maule earthquake

**Fig 3:**
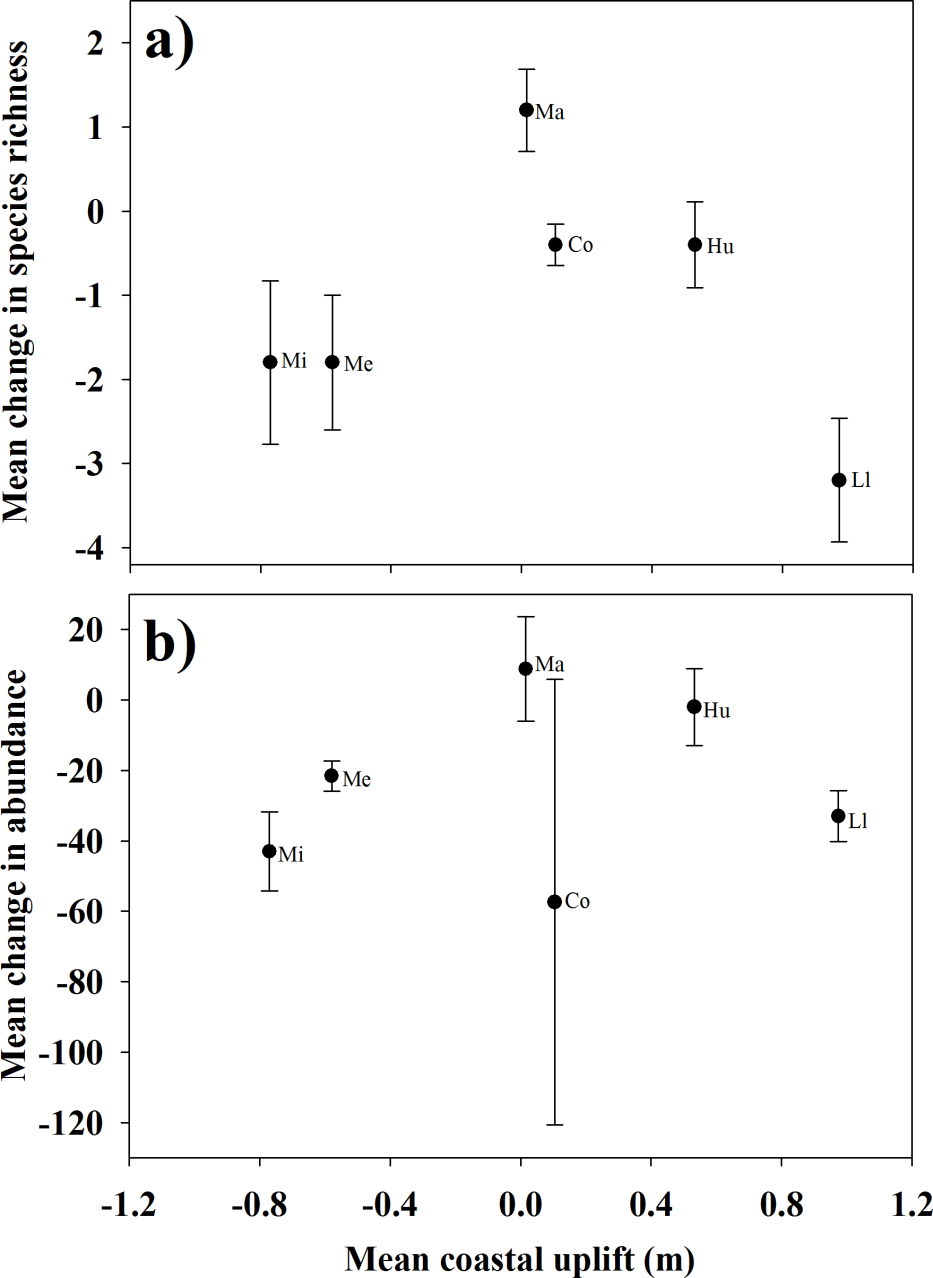
Coastal uplift and changes in species richness and total abundance from before (February 2010) to immediately after the earthquake (March 2010). Mean change was calculated by randomly pairing replicate transects from before- and after-earthquake samples and then averaging these values for each site. Values are given as mean ± 1 S.E.

Across the entire study region, species richness showed the strongest decrease from before to after the stroke at Llico, the site with the largest coastal uplift within the rupture area (Fig 3A). Mehuín and La Misión, both sites located in the control area, showed negative coastal deformations and also comparatively strong decreases in species richness (Fig 3A). Total abundance followed a similar pattern, with the exception of Cocholgüe (rupture area, Fig 3B). This site showed a nil coastal deformation and a comparatively large, but highly variable, mean decrease in total abundance (Fig 3B).

**Fig 4:**
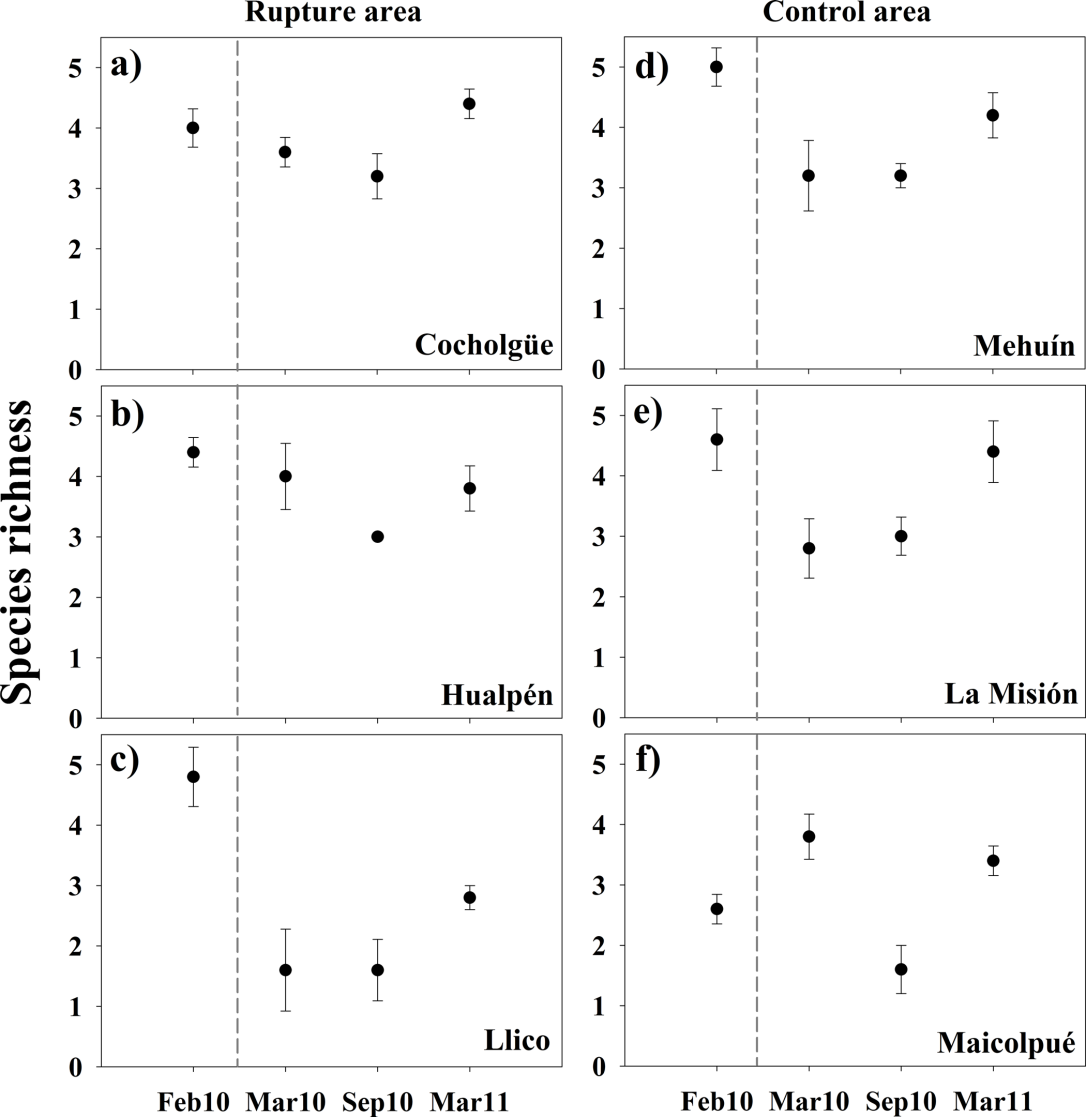
Number of invertebrate species in the intertidal sandy shores. Values are given for before (February 2010) and after (March 2010, September 2010, and March 2011) the earthquake and tsunami of February 27, 2010. Error bars denote ± 1 S.E.

For sites within rupture area the number of intertidal invertebrate species slightly decreased from February 2010 to September 2010 and increased during March 2011 (Fig 4A–4C). Particularly, Llico showed a sharp decrease in the mean number of species (Fig 4C). Among the control sites (Fig 4D–4F), we observed temporal fluctuations in species numbers of comparable magnitude to those observed in the rupture area. Mehuín and La Misión displayed the strongest variations in species richness within the control area, decreasing from February 2010 to March and September 2010—species richness increased in these sites in March 2011 (Fig 4D–4F). According to these patterns of variation in species richness, the PERMANOVA showed significant effects of Time × Site(Area), the interaction effect between Site and the temporal contrast (i.e. [Before vs. After] × Site(Area) in Table 1), and that of Time and the spatial contrast within the rupture area (i.e. Time × [L vs. C-H] term in Table 1).

**Table 1:** PERMANOVA for the spatiotemporal effects of the Maule earthquake on intertidal sandy-shore assemblages. The analyses were conducted on a Euclidean distance matrix for species richness and total abundance, and on a Bray-Curtis resemblance matrix of species abundances for community structure. The analysis was performed using Time (four sampling times: February 2010, March 2010, September 2010, and March 2011) and Area (two areas: Rupture and Control) as fixed factors, and Site (three sites located within each area) as random factor nested in Area. CV% = Components of variation.

		*Species richness*			*Total abundance*			*Community structure*		
Source of variation	*df*	_*pseudo*_*F*	*P*_*perm*_	*CV%*	_*pseudo*_*F*	*P*_*perm*_	*CV%*	_*pseudo*_*F*	*P*_*perm*_	*CV%*
Time	3	5.65	0.015	10.53	1.77	0.209	2.37	2.43	0.027	3.44
^a^[Before vs. After]	1	4.69	0.099	10.32	0.12	0.754	0.00	2.63	0.094	2.42
Area	1	0.01	0.924^c^	0.00	0.11	0.752^c^	0.00	1.00	0.431^c^	0.00
Site(Area)	4	9.11	<0.001	7.81	12.87	<0.001	25.23	22.29	<0.001	14.48
^b^[L vs C-H(Area)]	1	21.51	<0.001	14.17	0.01	0.916	0.00	51.18	<0.001	25.12
Time×Area	3	0.22	0.876	0.00	2.64	0.095	10.02	1.19	0.321	0.90
^a^[Before vs. After] × Area	1	0.28	0.633	0.00	1.65	0.265	2.17	0.74	0.603	0.00
Time×Site(Area)	12	3.52	<0.001	9.73	2.16	0.019	9.86	5.30	<0.001	11.70
^a^[Before vs. After] × Site(Area)	4	4.96	0.001	13.37	1.36	0.248	2.63	2.92	<0.001	5.84
^b^Time × [L vs. C-H(Area)]	3	6.37	0.001	14.84	1.49	0.222	5.21	12.24	<0.001	22.50
Residual	96			19.24			42.52			13.60

^a^Temporal independent contrast: before (February 2010) vs. after (March 2010-September 2010-March 2011) the Maule earthquake.

^b^Spatial independent contrast: Llico (Ll) vs. Cocholgüe-Hualpén (C-H) within the impacted area.

^c^P-value derived from Monte-Carlo simulations (P_MC_).

**Fig 5:**
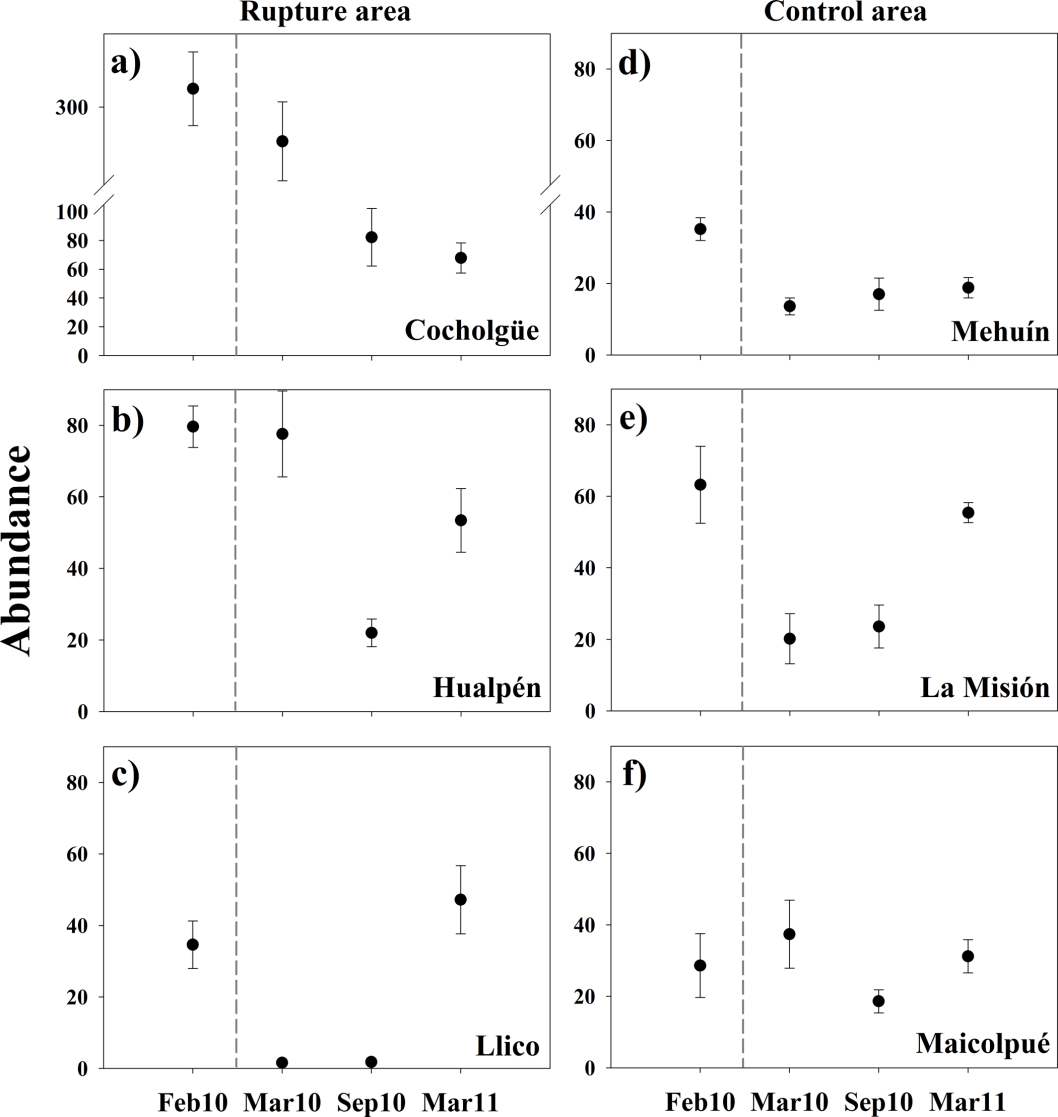
Total species abundance in the intertidal sandy shores. Values are given for before (February 2010) and after (March 2010, September 2010, and March 2011) earthquake and tsunami of February 27, 2010. Error bars denote ±1 S.E. *Note difference y-axis scale for Cocholgüe.

Total abundance strongly varied over times and among sandy shores (Fig 5). Within the rupture area (Fig 5A–5C), the sites showed a decrease of the abundances from February 2010 to September 2010, with a slight recovery during March 2011, which was not so evident for Cocholgüe (Fig 5A). In the control area, only Mehuín and La Misión decreased in abundance between February 2010 to March and September 2010 (Fig 5D and 5E); while Maicolpué remained homogeneous abundances during the study period (Fig 5F). The PERMANOVA indicated that the temporal trends in total abundance varied significantly among sites (i.e. Time × Site(Area) in Table 1). However, the spatial and temporal contrasts remained statistically non-significant for total abundance (Table 1).

**Fig 6:**
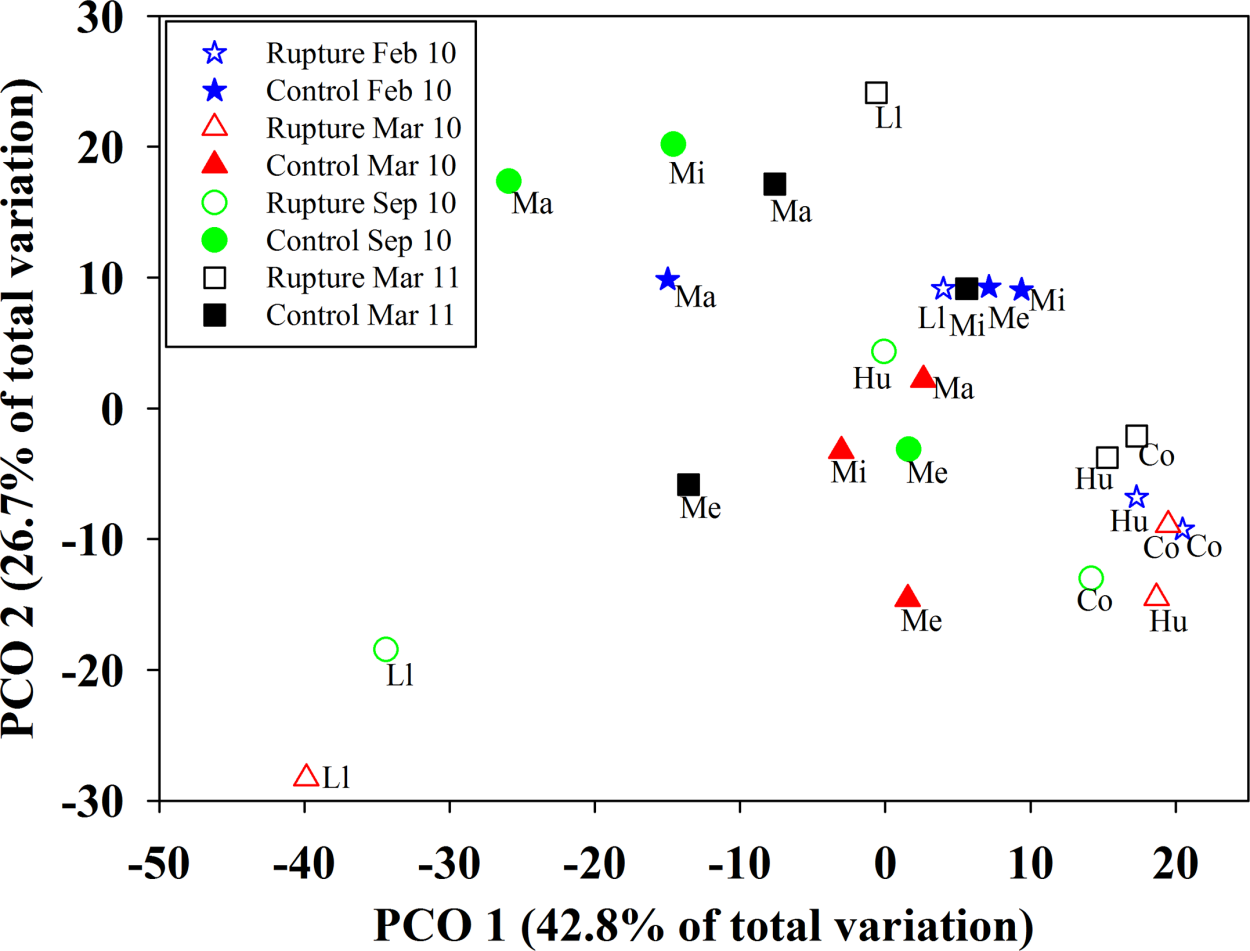
Principal Coordinates Analysis (PCoA) of the spatiotemporal patterns of community structure of six intertidal sandy shores. Ordination plot includes the rupture and control areas (empty and filled symbols, respectively) before (February 2010) and after (March 2010, September 2010, and March 2011) the earthquake and tsunami of February 27, 2010. Each symbol represent the centroid of five replicate transects deployed in each site and time.

The multivariate ordination showed that Llico performed the larger variation in community structure throughout time, as the centroids from March and September 2010 were well separated along the first PCO axis (Fig 6). The before-earthquake centroid (i.e. February 2010) of Llico remained in the main cloud of sites in the bivariate space (Fig 6). In addition, the sampling conducted 13 months after the earthquake lied within the main cloud, suggesting that the assemblage returned to a certain degree of the pre-disturbance structure. The samples from the control area remained at the centre of the ordination, with those samples from Maicolpué standing out along the second PCO axis (Fig 6).

According to the ordination plot, the PERMANOVA showed a significant Time×Site(Area) term, indicating that the temporal trends in effects of community-wide species abundances were spatially heterogeneous (Table 1). In addition, the significant interaction between the temporal contrast (i.e. [Before vs. After] × Site(Area)) supports the idea of a discernible effect of the earthquake on the structure of local assemblages. More specifically and within the rupture area, the temporal multivariate patterns of Llico were significantly different from those of the Cocholgüe-Hualpén centroid; that is, there was a significant interaction between Time and the spatial contrast (i.e. Time × [L vs. C-H(Area)] term in Table 1). The statistical terms that included the spatial contrast accounted for the highest multivariate components of variation (22.50%). For community structure, as well as for species richness and total abundance, the residual term contributed a high proportion of variability, hinting for a high spatial patchiness at the local scale. The overall multivariate similarity between before- and after-earthquake samples slightly increased over time. Between February 2010 and March 2010 samples, similarity was of 76.92%, between February 2010 and September 2010 was of 79.19%, and between February 2010 and March 2011 was of 87.71%. Llico, on the other hand, showed a steeper increase in similarity. Community structure between February and March 2010 showed 27.88% similarity, between February and September 2010 a 31.57% similarity, and between February 2010 and March 2011 reached up to 49.19% similarity.

Four species accounted for most of the total abundance across sites and sampling times (Table 2). The temporal patterns of these taxa were highly variable between sites. At Llico, the mid-intertidal cirolanid isopod *Excirolana hirsuticauda* and the high-intertidal talitrid amphipod *Orchestoidea tuberculata* (*Eh* and *Ot* in Table 2, respectively) occurred in February 2010, showed a general pattern of near-zero abundances during March and September 2010, and a further increase in March 2011. The low-intertidal mole crab *Emerita analoga* (*Ea* in Table 2) remained with zero abundance in the end of the study in March 2011. Within the rupture area, the polychaete *Euzonus heterocirrus* (*Eu* in Table 2) was relatively abundant at Cocholgüe and Hualpén, but nearly absent at Llico; in the control area, this species was also scarce.

**Table 2:** Mean abundances (± 1 SD) for the four most abundant species in the intertidal sandy shores. Abundances values are given for the rupture and control areas registered before (February 2010) and after (March 2010, September 2010 and March 2011) the Maule earthquake (27 February 2010). The listed species are the anomuran crab *Emerita analoga* (*Ea*, low intertidal), the cirolanid isopod *Excirolana hirsuticauda* (*Eh*, mid-intertidal), the talitrid amphipod *Orchestoidea tuberculata* (*Ot*, high intertidal), and the ophelid polychaete *Euzonus heterocirrus* (*Eu*, low/mid-intertidal).

Site	Species	February 2010	March 2010	September 2010	March 2011
Cocholgüe	*Ea*	291.2 (91.2)	243.2 (87.7)	72.8 (45.2)	31.0 (12.9)
	*Eh*	7.6 (3.4)	5.6 (4.0)	2.4 (2.1)	11.4 (4.4)
	*Ot*	3.0 (1.9)	5.6 (3.1)	6.4 (3.1)	7.4 (6.4)
	*Eu*	18.0 (12.2)	8.2 (8.6)	0.6 (0.9)	17.6 (13.3)
Hualpén	*Ea*	49.0 (17.9)	62.4 (25.2)	5.8 (2.9)	19.4 (16.8)
	*Eh*	10.2 (5.6)	2.0 (1.2)	13.0 (6.2)	14.0 (9.1)
	*Ot*	3.4 (2.4)	9.6 (2.5)	3.2 (1.9)	15.2 (4.2)
	*Eu*	16.6 (11.3)	3.0 (3.1)	0.0 (0.0)	4.6 (3.0)
Llico	*Ea*	14.6 (12.6)	0.4 (0.5)	1.0 (0.7)	0.0 (0.0)
	*Eh*	14.2 (6.7)	0.0 (0.0)	0.4 (0.5)	33.8 (24.1)
	*Ot*	3.8 (3.1)	0.0 (0.0)	0.0 (0.0)	11.4 (6.7)
	*Eu*	0.0 (0.0)	0.20 (0.5)	0.0 (0.0)	0.0 (0.0)
Mehuín	*Ea*	6.8 (5.3)	7.0 (3.2)	7.6 (8.6)	2.8 (3.1)
	*Eh*	9.4 (3.6)	0.8 (0.8)	3.2 (2.2)	5.2 (5.5)
	*Ot*	11.6 (8.8)	5.2 (4.1)	5.6 (2.9)	2.0 (2.5)
	*Eu*	0.2 (0.4)	0.0 (0.0)	0.0 (0.0)	0.0 (0.0)
La Misión	*Ea*	11.0 (4.3)	1.8 (1.9)	1.0 (1.7)	4.2 (1.6)
	*Eh*	17.0 (11.8)	2.6 (3.2)	14.6 (4.8)	13.6 (5.4)
	*Ot*	32.4 (12.5)	15.6 (11.0)	6.2 (11.8)	35.6 (10.2)
	*Eu*	0.0 (0.0)	0.0 (0.0)	0.0 (0.0)	0.0 (0.0)
Maicolpué	*Ea*	3.4 (2.2)	6.8 (5.9)	0.6 (0.9)	1.4 (1.5)
	*Eh*	24.4 (20.4)	27.2 (17.5)	17.8 (7.1)	27.8 (10.1)
	*Ot*	0.2 (0.4)	1.4 (1.1)	0.0 (0.0)	1.0 (0.7)
	*Eu*	0.0 (0.0)	1.8 (1.6)	0.0 (0.0)	0.0 (0.0)

## Discussion

Our results showed concurrent patterns of change in beach profiles and sandy-beach assemblages from before to after the Chile 8.8 mega-earthquake. In agreement with recent geomorphological studies [7,19], we observed that the deformation of beach profiles following the earthquake was heterogeneous along the rupture area. Llico, a site located at the centre of the rupture area [7] and impacted hardest by the after-earthquake tsunami, showed the strongest changes in beach profile and, thus, in species richness and community structure. The multivariate analyses also suggested that the effect of the earthquake on community structure significantly decreased 13 months after the mega-earthquake took place. Therefore, these results support our hypothesis of spatially heterogeneous effects of the Maule earthquake and tsunami on the diversity and structure of local sandy beach communities. On the following lines, we propose testable hypotheses to explain these patterns in the light of current ideas about the role of disturbances in structuring sandy-shore communities. With this, we hope to stimulate further experimental research to understand how these dynamic systems respond to present and projected disturbance regimes.

Wave-exposed sandy shores are characterised by wide and persistent changes in shore morphology and beach profiles. In particular, sandy shores show significant depressions of beach profile during autumn and winter (e.g. [6,22]). According to our results, only the patterns of those sites located in the control areas conformed to this general pattern of seasonal erosion. The exception in the control area was Maicolpué (Ma), site that showed a delayed downward movement of the beach profile in September 2010. This lack of normal seasonal changes in beach profiles has been explained by the effect of manmade coastal defenses that significantly reduce the natural variation of sandy beaches [6]. On the other hand, the sites located in the rupture area showed very unusual patterns of variation in beach profile after the earthquake took place—the three locations performed different degrees of coastal uplift contrary to the regular winter erosion. As in the control area, the small albeit still positive uplift observed in Cocholgüe can be explained as a response to manmade coastal structures that buffer sediment movement [6]. Therefore, there is a very high probability that the unusual coastal uplift observed in the rupture zone was a consequence of the Maule earthquake that took place in February 2010.

In our study, Llico showed the strongest changes in the profile of the beach and also in species richness and community structure. Unfortunately, we did not count with long-term time series of species abundances at this site. Nevertheless, our spatially extensive design allows us to determine that the impacted site harboured the lowest pre-stroke population numbers in the rupture zone. Low population densities can negatively affect different components of individual fitness, which can scale up to the demography of the entire population [23]. Dominant taxa in these ecosystems, such as mole crabs, cirolanid isopods, and talitrid sandhoppers, perform mating as a reproductive strategy (e.g. [24,25]). Such strategy can be very sensitive to variations in population numbers, because low numbers can reduce the probability of encounter between females and males [26]. Theoretical work suggests that increased extinction risks in species with small population abundances and densities can lead to community-wide instability (e.g. [27,28]), which might explain the high temporal variability observed at the community level in the impacted site. The variation in population-level attribute, therefore, could be a pivotal element the community-wide resistance to disturbances—and thus stability—of intertidal sandy shores.

The effects of the mega-disturbance on emergent community properties significantly decreased after 13 months. This potential community-wide recovery was mostly accounted for by the increase in abundance of *E*. *hirsuticauda* and *O*. *tuberculata*, which show adaptations to dynamic environmental conditions and unpredictable impacts. For example, sandhopper’ adaptations can include, among others, rapid escape reactions and horizontal orientation toward optimal zones of the beach [29–32]. In southern Chile, *O*. *tuberculata* and *E*. *hirsuticauda* show different patterns of movement and habitat use in the beach, which likely prevents interspecific competitive interactions [33]. Interesting, some areas within the impacted sites (i.e. areas armoured with manmade structures) showed a rapid colonisation of sandhoppers and isopods right after the earthquake, generating a local spatial mosaic of population abundances [33]. Therefore, the ability of sandhoppers and beach isopods to actively move between different zones of the beach could have mediated the rapid increase in population numbers after the disturbance took place. In a more general context, the movement of organisms among habitat patches can have significant effects on the stability of the entire meta-community, as suggested by theoretical models [34] and manipulative field experiments (e.g. [35]). Manipulative experiments, in addition to the assessment of dispersal strategies of dominant species (e.g. [36]), should be conducted in order to test the effects of varying levels of species mobility on the resilience and elasticity of sandy beach communities.

Contrarily to the peracarid species, the abundance of the mole crab *E*. *analoga* showed virtually no recovery after the earthquake by March 2011. Perhaps, the significant and persistent changes in the profile of the beach, likely triggered by the Maule earthquake, resulted in more prohibitive environmental conditions for the reestablishment of *E*. *analoga* immigrants in Llico. The tsunami waves deposited large amounts of sand on intertidal sandy shores and also generated significant changes in the granulometry of beaches to coarser grain sizes [22]. Morphodynamic beach properties, including granulometry, have been shown to significantly account for the probability of occurrence of benthic communities in southern Chile and elsewhere [37–39]. In particular, burial rate of *E*. *analoga* is slower in beaches with coarser grain sizes [40], which can reduce the chances for survival of new recruits after they are swept away by waves. Nevertheless, this species can show morphological adaptations that can improve the burrowing performance of local subpopulations [40]. Testing the effect of morphodynamic beach properties on the capacity of recovery of local population numbers would require the combination of removal and transplant experiments in order to account for the influence of local adaptations.

In summary, the beyond-BACI sampling design allowed us to detect significant changes in species richness and community structure from before to after the occurrence of the Maule earthquake and concomitant tsunami. The disturbance seemed to have a localised effect on the community dynamics, at least, within the southern-central section of the earthquake rupture area. The spatially extensive analysis showed a high background variability in habitat physical properties and ecological communities, which, if not accounted for, could well confound the effect of the additional disturbances triggered by the earthquake and tsunami. Hopefully, our analyses will stimulate further experimental research on the effects of individual- and population-level properties on the response of sandy beach communities to interacting sources of disturbances. Untangling the effects of different kind of disturbances on community dynamics is essential to develop predictive models of community stability in the face of global-scale environmental changes.

## Supporting Information

S1 Table Raw data matrix of intertidal sandy-shores species abundances estimated at six sites along the southern Chilean coast before and after the Maule earthquake of 27 February 2010. (XLSX)
